# Peroral cholangioscopy-guided basket extraction of intrahepatic bile duct stones, using a novel thin cholangioscope under balloon enteroscopy, in a patient with Roux-en-Y anatomy

**DOI:** 10.1055/a-2512-5414

**Published:** 2025-01-28

**Authors:** Yuki Tanisaka, Shomei Ryozawa, Masafumi Mizuide, Akashi Fujita, Ryuhei Jinushi, Ryuichi Watanabe, Ryo Sato

**Affiliations:** 1 183786 Gastroenterology, Saitama Medical University International Medical Center, Hidaka, Japan

Endoscopic stone extraction using a balloon enteroscope in patients with a Roux-en-Y anastomosis is challenging due to the difficulty in aligning the axis between retrieval devices and stones, which often results in failure of stone capture 1 2 . Recently, it has been reported that a novel thin cholangioscope (EyeMax; Micro-Tech, China), with a length of 219 cm and a diameter of 9 Fr, enables peroral cholangioscopy (POCS)-guided procedures using a balloon enteroscope with a 3.2-mm forceps channel ( Fig. 1 ) 3 4 . Furthermore, it also enables POCS-guided basket extraction. We report a case of successful POCS-guided basket extraction of intrahepatic bile duct stones using a novel thin cholangioscope under balloon enteroscopy in a patient who had undergone a Roux-en-Y procedure.

**Fig. 1:**
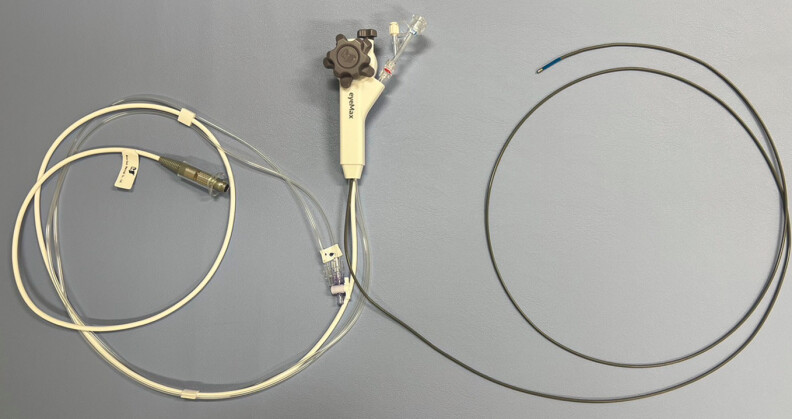
Thin cholangioscope (EyeMax; Micro-Tech, China), length 219 cm, diameter 9 Fr.

A 68-year-old woman who had undergone hepaticojejunostomy with Roux-en-Y construction was referred to us. Computed tomography revealed stones in the intrahepatic bile duct ( Fig. 2 ). Although the previous hospital had attempted stone extraction, the stones could not be captured using the basket catheter. Therefore, we performed endoscopic retrograde cholangiopancreatography (ERCP) using a short-type single-balloon enteroscope (SIF-H290; Olympus, Japan) with a working length of 152 cm and a working channel of diameter 3.2 mm 1 ( Video 1 ). Cholangiography revealed stones in the intrahepatic bile duct ( Fig. 3 ). Subsequently, POCS was performed using a thin cholangioscope, revealing stones in the intrahepatic bile duct ( Fig. 4 ). Since the stones were considered difficult to capture, POCS-guided basket extraction was attempted using a retrieval basket (SpyGlass Retrieval Basket; Boston Scientific, USA) ( Fig. 5 **a** ), that could be inserted through the thin cholangioscope. The stones were successfully captured under cholangioscopic visualization ( Fig. 5 **b, c** ). Finally, complete stone extraction was confirmed using the cholangioscope ( Fig. 5 **d** ).

**Fig. 2:**
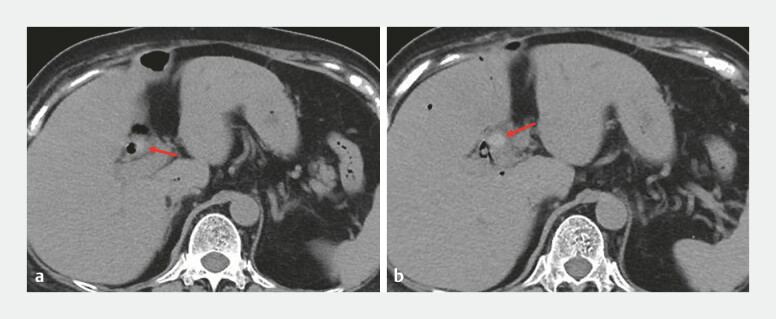
Computed tomography revealed stones (arrows) in the intrahepatic bile duct.

**Fig. 3:**
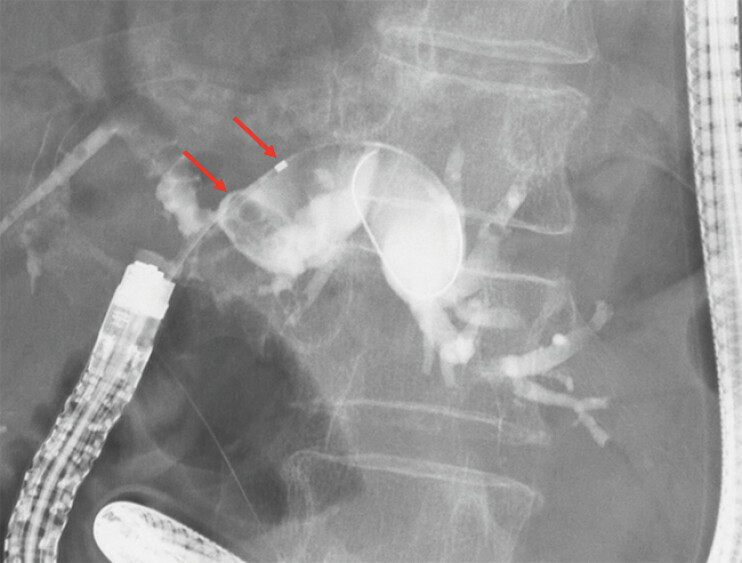
Cholangiography revealed stones (arrows) in the intrahepatic bile duct.

**Fig. 4:**
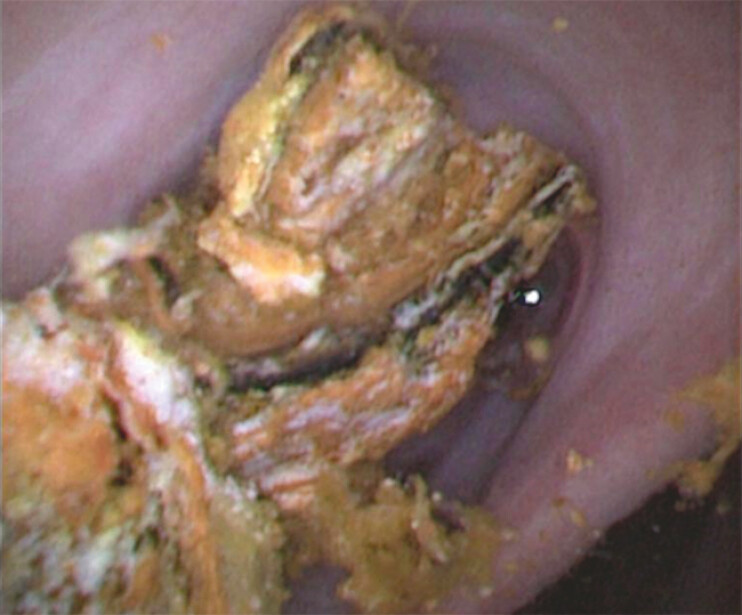
Cholangioscopic view of stones in the intrahepatic bile duct.

**Fig. 5:**
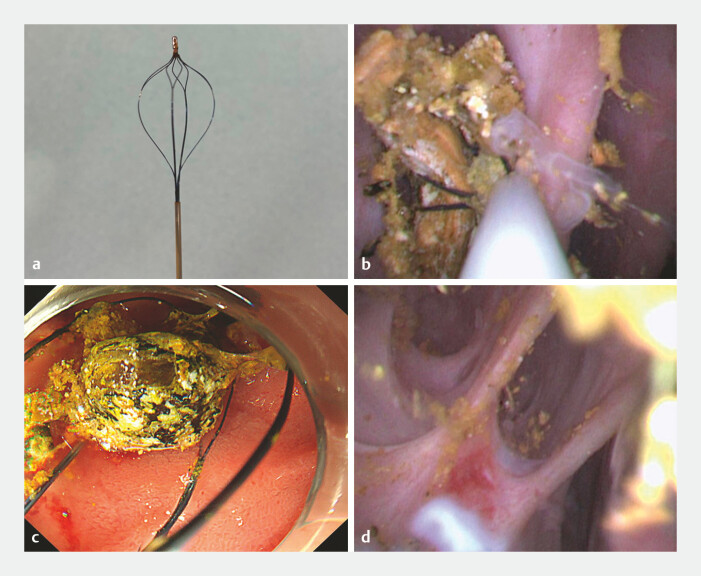
Cholangioscopic and endoscopic findings. **a** Retrieval basket (SpyGlass Retrieval Basket; Boston Scientific, USA) that could be inserted through the thin cholangioscope. **b, c** The stones were successfully captured under cholangioscopic visualization. **d** Complete stone extraction was confirmed using the cholangioscope.

Peroral cholangioscopy-guided basket extraction of intrahepatic bile duct stones, using a novel thin cholangioscope under balloon enteroscopy, in a patient with Roux-en-Y anatomy.Video 1

POCS-guided basket extraction is beneficial for capturing difficult stones, as POCS allows for direct visualization of stones 5 . This thin cholangioscope enables POCS-guided basket extraction under balloon enteroscopy in patients with Roux-en-Y anatomy.

Endoscopy_UCTN_Code_TTT_1AR_2AH

## Notes

Endoscopy E-Videos https://eref.thieme.de/e-videos
*E-Videos* is an open access online section of the journal *Endoscopy* , reporting on interesting cases and new techniques in gastroenterological endoscopy. All papers include a high-quality video and are published with a Creative Commons CC-BY license. Endoscopy E-Videos qualify for HINARI discounts and waivers and eligibility is automatically checked during the submission process. We grant 100% waivers to articles whose corresponding authors are based in Group A countries and 50% waivers to those who are based in Group B countries as classified by Research4Life (see: https://www.research4life.org/access/eligibility/ ). This section has its own submission website at https://mc.manuscriptcentral.com/e-videos .
